# Multi-breed genome-wide association study reveals novel loci associated with the weight of internal organs

**DOI:** 10.1186/s12711-015-0168-7

**Published:** 2015-11-17

**Authors:** Yuna He, Xinjian Li, Feng Zhang, Ying Su, Lijuan Hou, Hao Chen, Zhiyan Zhang, Lusheng Huang

**Affiliations:** National Key Laboratory for Swine Genetics, Breeding and Production Technology, Jiangxi Agricultural University, Nanchang, 330045 China

**Keywords:** GWAS, Pig, Haplotype GWAS, Organ weight

## Abstract

**Background:**

Recently, many genome-wide association studies (GWAS) have been conducted to understand the genetic architecture of economic important traits in farm animals. Pig is widely used as a biomedical animal model for its similarity with humans in terms of organ formation and disease mechanisms. Moreover, understanding the mechanisms that underlie the development of internal organs will impact the productive potential of pigs. Our aim was to uncover new single nucleotide polymorphisms (SNPs) associated with the weight of internal organs and carcass and also potential candidate genes.

**Methods:**

We performed GWAS for the weight of heart, liver, spleen, kidney and carcass on five pig populations (White Duroc × Erhualian F_2_ intercross, Sutai population, Laiwu population, Erhualian population and commercial population, for a total of 2650 individuals). Genotype data was produced using the PorcineSNP60 Beadchip array. After quality control, the data was used for association tests under a general linear mixed model. Population stratification was adjusted by including a random polygenic effect based on a matrix of genotypic relationships. A meta-analysis of our GWAS datasets was conducted by summing up the Chi square values across breeds, with the degrees of freedom of the Chi square distribution equal to the effective number of breeds.

**Results:**

Thirty-nine quantitative trait loci (QTL) located on 15 chromosomes were identified by the single-population GWAS at the suggestive level. Among these, nine QTL surpassed the 5 % genome-wide significance threshold, including four for heart weight on SSC (*Sus scrofa* chromosome) 2, 4, 7 and 10, two for liver weight on SSC7, two for spleen weight on SSC5 and SSC7 and one for carcass weight on SSC11. The QTL on SSC7 showed pleiotropic effects for heart, liver and spleen weights in the F_2_ population. In addition, two QTL were detected in several populations, including one on SSC2 for heart weight in the F_2_ and Sutai populations and one on SSC7 for liver weight in the F_2_ and Laiwu populations. The meta-analysis detected four novel QTL on SSC1, 3, 8 and 16 for carcass weight.

**Electronic supplementary material:**

The online version of this article (doi:10.1186/s12711-015-0168-7) contains supplementary material, which is available to authorized users.

## Background

With the development of next-generation sequencing technologies [1, 2] and sophisticated statistical methods, it has become possible to identify the genetic basis of more and more economic important complex traits. For example, it has been shown that the genes *insulin*-*like growth factor 2* (*IGF2*) and *ryanodine receptor 1* (*RYR1*) are associated with an increase in muscle mass and a reduction in backfat thickness [3] and with pale, soft and exudative meat [4], respectively. Implementation of marker-assisted selection (MAS) in breeding schemes has greatly benefited the pig industry [5]. In recent years, because of the advent of high-density single nucleotide polymorphisms (SNPs) genotyping panels, genome-wide association studies (GWAS) have been widely used to identify candidate genes and quantitative trait loci (QTL) for a number of complex traits in humans, animals and plants [6–9]. Compared to traditional QTL mapping strategies [10], GWAS do not require pedigree information or any prior assumption on the fixation of QTL alleles in the founder populations. These advantages make GWAS one of the most popular strategies for the study of complex diseases in humans or of economic important traits in farm animals [11].

Previous studies have shown that pigs and humans share similar characteristics in terms of body weight, physiological features, organ formation and mechanisms of disease infection [12]. Using the pig as a biomedical model has led to significant progress in the study of atherosclerosis and diabetes [13]. The pig is also considered as the most optimal donor of internal organs (e.g. heart) for humans [12, 14]. Deciphering the genetic architecture of complex traits such as the weight of internal organs in the pig will help to better understand the pathogenesis of human diseases associated with internal organs and to develop new gene therapy methods. Body weight is the sum of fat mass, internal organ mass, muscle mass and skeleton mass. Each of these components has its own developmental process and gene expression profile [15].

To date, the genetic architecture that underlies the weight of internal organs is not completely known. To our knowledge, most studies published on QTL associated with the weight of internal organs in pigs were based on QTL mapping strategies [16] and few used GWAS [17]. Furthermore, most of these studies focused mainly on one breed or on intercross populations and, thus, the results only reflect a small proportion of the genetic mechanisms that underlie these complex traits and provide little information on the genetic homologies and differences between breeds. These limitations have hindered the progress in the fine-mapping and detection of additional QTL for these traits.

In this study, we conducted a GWAS on five pig populations that were genotyped with the PorcineSNP60 Beadchip array. Furthermore, a meta-analysis was performed by combining the five populations. We also attempted to highlight the genetic homologies and differences that underlie these complex quantitative traits between breeds and populations.

## Methods

### Animals and sample collection

Animal care and tissue collection procedures followed the guidelines established by the Ministry of Agriculture of China. The ethics committee of Jiangxi Agricultural University specifically approved this study.

Experimental animals were from five populations i.e.: (1) a White Duroc × Erhualian F_2_ intercross that derived from two White Duroc founder boars and 17 Chinese Erhualian founder sows (consisting of a sub-population of Chinese Taihu pigs) and comprised 1912 F_2_ pigs in six batches; (2) a Sutai population, which is a Chinese synthetic pig line that was originally generated from Chinese Taihu and Duroc pigs; the current population was generated by over 18 generations of artificial selection; in our study, we used 460 Sutai pigs from five sires and 60 dams; (3) a population of 316 Laiwu pigs; (4) a population of 334 Erhualian pigs that originated both from Chinese native purebred populations and from farms in Laiwu (Shandong province) and Changzhou (Jiangsu province), respectively; and (5) a commercial population that was a three-way-cross breed (Duroc × Large White × Yorkshire or DLY), for which 610 samples were collected. For the F_2_ and Sutai populations, fattening pigs were raised under consistent indoor conditions and were slaughtered at the age of 240 days. Only 1028 of the 1912 F_2_ individuals were slaughtered and recorded for phenotypic traits evaluated in this study; the other individuals were used for a study on male/female reproduction traits. Because the Chinese native breeds have a slower growth rate, they were fed ad libitum rather than on restricted feeding, which varied with age as for commercial breeds, and they were slaughtered at the older age of 300 days. DLY pigs were slaughtered at a weight of about 90 kg, which corresponds to about 180 days of age. Immediately after slaughter, we measured the weights of the heart, liver, spleen, kidney and carcass for the five populations except that kidney weight was not recorded for DLY pigs. Ear tissue was collected and stored in 75 % ethyl alcohol for DNA extraction.

### Genotyping and quality control

For each animal, we extracted genomic DNA from ear tissue using a standard phenol/chloroform method. DNA was diluted to a final concentration of 50 ng/µL. Genotyping was performed using the PorcineSNP60 Beadchip array (Version 1 for the F_2_ and Sutai populations and Version 2 for the Laiwu and Erhualian pigs and the commercial population) according to the manufacturer’s protocol. Signal intensities from the Illumina iScan were normalized and genotypes were called using the Genome Studio software. Quality control procedures were carried out using PLINK v1.07 software [18], separately for each population. Briefly, samples with a call rate greater than 0.95 and a Mendelian error rate less than 0.05, and SNPs with a call rate greater than 0.95, a minor allele frequency (MAF) higher than 0.05 and a Mendelian error rate less than 0.05 were kept for further analysis. After quality control, 45,242, 46,347, 41,264, 30,410 and 51,668 SNPs and 928, 436, 317, 333 and 610 individuals remained for single-population GWAS for the F_2_, Sutai, Laiwu, Erhualian and DLY populations, respectively. In total, 45,962, 45,962, 45,962, 44,858 and 46,629 SNPs were retained for the meta-analysis that included heart weight, liver weight, spleen weight, kidney weight and carcass weight.

### Phenotype analyses

Correlations between traits and simple statistics were summarized using R software. Heritability was estimated using the polygenic function of the R package GenABEL [19]. We then used the likelihood ratio test statistic (LRT) = −2ln(L0/L1) to test for the presence of heritable variance against the null hypothesis of no heritable variance, where L0 and L1 represented the likelihood values of the reduced (1) and full (2) models, respectively [20].

### Single-population GWAS and meta-analysis

Association between each SNP and the phenotypic traits in each population was tested by fitting an additive model under a generalized linear mixed model using the GenABEL package [19], separately for each SNP and population. The model adjusted for population stratification by including a random polygenic effect, with the corresponding variance–covariance matrix proportionate to pairwise genome-wide identity-by-state. This model is denoted as:

$${\mathbf{y}} = {\mathbf{X}}{\text{b}} + {\mathbf{s}}{\upalpha } + {\mathbf{Z}}{\text{u}} + {\mathbf{e}},$$

where **y** is the vector of phenotypes, b is the estimator of fixed effects including sex, batch and body weight, α is the SNP substitution effect and u is the random additive genetic effect following a multinomial distribution u ~ N(0, **G**σ_α_^2^) where **G** is the individual–individual similarity kinship matrix estimated by whole-genome SNPs as described in Eding et al. [21], and σ_α_^2^ is the polygenetic additive variance. **X**, **Z** and **s** are the incidence matrices (vector) for b, u and α. **s** was coded as 0, 1, or 2 corresponding to the three genotypes 11, 12, and 22 of the tested SNP. **e** is a vector of residual errors with a distribution of N(0, **I**σ_e_^2^). Since phenotypes were not measured at consistent ages and conditions between populations, trait distributions differed between populations and the estimated SNP effects could not be integrated directly. The Chi square, *p* value and allele effect of each SNP were calculated with the GenABEL packages. A meta-analysis to test for association of a SNP with phenotype across populations was conducted by computing:

$$S_{l} = \sum\limits_{i = 1}^{k} {(\chi_{li}^{2} } ),$$

where *S*_*l*_ is the sum of Chi square values from the single-population analyses for population *i* to population *k* at SNP *l*. Under the null hypothesis of no association, this summed score (*S*_*l*_) is approximately distributed as a Chi square with *k* degrees of freedom, where *k* is equal to the number of populations for which the SNP is analyzed and that contribute to the test statistic [22]. This method has the advantage that it does not make assumptions on whether the estimated effects of the SNPs are consistently negative or positive across populations. Thus, for a SNP with an allele B that has a negative effect in population 1 and a positive effect in population 2, our method will detect a significant effect, while a joint analysis using standardized data would not, unless an interaction between SNP and population is fitted.

For both single-population GWAS and the meta-analysis, suggestive and genome-wide significance thresholds [23] were determined by Bonferroni correction, which was defined as 1/N and 0.05/N, where N is the number of tested SNPs. Tested SNPs were positioned on pig chromosomes according to the current assembly of the pig genome (build 10.2) [24]. A cluster of significant SNPs within a linkage disequilibrium (LD) block was treated as a single significant QTL. When the distance between two consecutive genome-wide significant SNPs was greater than 10 Mb, they were considered as representing two separate QTL [25].

The phenotypic variance explained by the top SNP was calculated with the R software as (V_reduce_ − V_full_)/V_phe_, where V_full_ and V_reduce_ are the residual variances of models with and without the SNP effect and V_phe_ is the phenotypic variance.

## Results

### Phenotype statistics

Means, standard errors and estimated heritabilities (*h*^2^) for the weights of the four analyzed internal organs and carcass are in Table 1 and estimated correlations and 95 % confidence intervals for these traits are in Table S1 (see Additional file 1: Table S1). Mean weights of heart, liver, spleen and carcass were largest for DLY pigs and smallest for Laiwu (or) Erhualian pigs. Phenotypic correlations were highest between carcass weight and heart weight and lowest between heart weight and spleen weight for all populations except for the Laiwu breed. In most cases, estimated heritabilities for these five traits ranged from 0.3 to 0.56, which suggests that there is considerable genetic contribution to the weights of internal organs and carcass.

**Table 1:** Descriptive statistics for the traits in pigs from five populations

Population	Trait	Heart weight	Liver weight	Spleen weight	Kidney weight	Carcass weight
F2	Nb individuals	924	923	920	923	928
	Mean ± SD	0.34 ± 0.07	1.39 ± 0.27	0.13 ± 0.03	0.28 ± 0.06	34.65 ± 7.19
	*h* ^*2*^	0.56**	0.40**	0.54**	0.35**	0.37**
Sutai	Nb individuals	436	436	434	436	436
	Mean ± SD	0.28 ± 0.06	1.11 ± 0.24	0.15 ± 0.04	0.25 ± 0.04	50.98 ± 13.01
	*h* ^*2*^	0.41**	0.49**	0.56**	0.41**	0.41**
Laiwu	Nb valid individuals	317	302	317	317	317
	Mean ± SD	0.28 ± 0.04	1.16 ± 0.21	0.12 ± 0.05	0.23 ± 0.04	61.40 ± 12.05
	*h* ^*2*^	0.25**	0.38**	0.34**	0.52**	0.34**
Erhualian	Nb valid individuals	332	312	333	332	333
	Mean ± SD	0.31 ± 0.05	1.24 ± 0.24	0.17 ± 0.04	0.27 ± 0.06	66.2 ± 11.40
	*h* ^*2*^	0.51**	0.36**	0.43*	0.44**	0.45**
DLY	Nb valid individuals	609	610	604	0	609
	Mean ± SD	0.40 ± 0.05	1.68 ± 0.21	0.18 ± 0.04	–	84.95 ± 8.40
	*h* ^*2*^	0.5**	0.53**	0.46**	–	0.22*

*Nb* number, *h* ^2^ heritability estimate

* Significance level (*p* < 0.01)

** Extreme significance level (*p* < 0.001)

### GWAS results

**Fig. 1:**
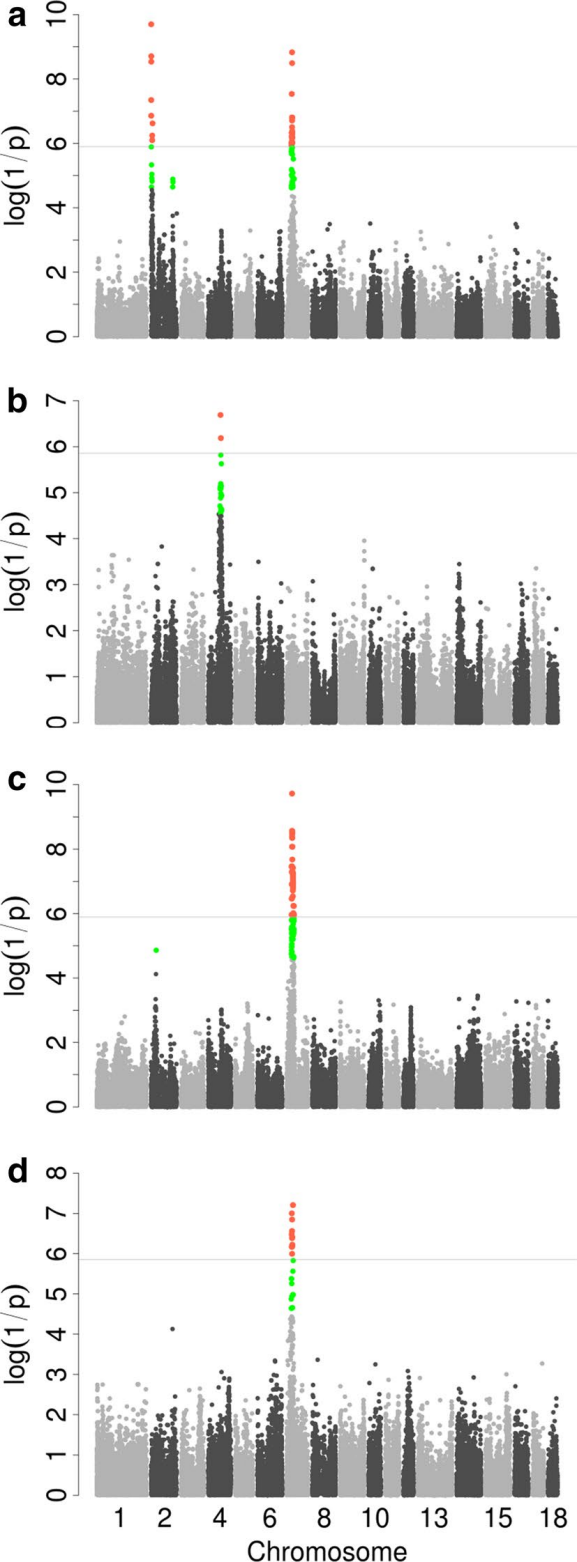
Single-population GWAS results for heart weight and liver weight. Manhattan plots for heart weight in F_2_ (**a**) and Laiwu pigs (**b**), and for liver weight in F_2_ (**c**) and Laiwu pigs (**d**). In the Manhattan plots, negative log_10_ *p* values of the SNPs were plotted against their genomic positions; the *red* and *green dots* represent SNPs that exceeded the suggestive and 5 % genome-wide significance thresholds, respectively; *solid lines* indicate the 5 % genome-wide Bonferroni-corrected threshold

We identified 39 QTL distributed on 15 chromosomes that satisfied the suggestive significance threshold used for the traits studied (see Additional file 2: Table S2). For the F_2_ population, we identified nine QTL that reached the suggestive significance level, of which three exceeded the genome-wide significance level (Table 2; Fig. 1). For heart weight, two genome-wide QTL were identified: one on SSC2 between 0.2 and 0.9 Mb, and one on SSC7 between 31 and 48.1 Mb, each of these regions including 17 and 50 significant SNPs, respectively. For liver weight, one QTL was detected on SSC7 between 31.0 and 47.2 Mb, a region that comprised 95 significant SNPs. Interestingly, 43 SNPs on SSC7 were significant both for the QTL for heart weight and the QTL for liver weight. The top SNP, rs80935535 (34.8 Mb) was also the same for these two significant QTL, accounting for 13.2 % of the phenotypic variance for both heart and liver weights. This suggests that these two phenotypes may share a common genetic mechanism.

**Table 2:** Genome-wide significant QTL identified by GWAS for the weight of four internal organs and carcass in five populations

Trait	Chr	Population	Nb_snp_	Top SNP	Position (bp)	*p* value	Var (%)	Candidate gene
Heart weight	2	F_2_	17	rs81318741	920,370	2.00E−10	8.6	*IGF2*
	4	Laiwu	20	rs80991149	77,161,841	2.05E−07	8.7	
	7	F_2_	25	rs80935535	34,803,564	1.48E−09	13.2	*GRM4, HMGA1*
	10	DLY	1	rs81334236	32,207,568	9.05E−07	3.2	
Liver weight	7	F_2_	95	rs80935535	34,803,564	1.91E−10	13.2	*GRM4, HMGA1*
		Laiwu	25	rs80917438	40,398,610	6.20E−08	10.7	
Spleen weight	5	DLY	1	rs81303231	95,090,653	3.54E−07	5.5	
	7	Erhualian	1	rs80965843	97,889,360	3.05E−07	2.6	
Carcass weight	11	Sutai	1	rs81000302	48,894,442	9.95E−08	16.1	

*Chr* chromosome number,* Nb*_*snp*_ number of SNPs that reached the suggestive significance level,* Var (%)* % of phenotypic variance explained by the top SNP

Two genome-wide significant QTL and 12 suggestive QTL were identified for the Laiwu population (Table 2; Fig. 1). Of the two genome-wide significant QTL, a QTL for heart weight was located on SSC4, which included 20 SNPs, and a QTL for liver weight was located on SSC7, which included 24 SNPs. The QTL associated with liver weight on SSC7 comprised six significant SNPs that were shared between the F_2_ and Laiwu pig populations, which suggests that the variant responsible for the QTL effect on liver weight is the same in both populations.

For the other three populations (Erhualian, DLY commercial pigs, Sutai), several QTL were detected: (1) one genome-wide significant QTL at 97.9 Mb on SSC7 for spleen weight and three suggestive QTL, including one on SSC7 for heart weight and two on SSC7 and SSC12 for carcass weight in Erhualian pigs; six QTL in DLY commercial pigs, including two genome-wide significant QTL associated with spleen weight (SSC5 at 95.1 Mb) and heart weight (SSC10 at 32.2 Mb); and eight QTL in Sutai pigs, of which one achieved genome-wide significance level for carcass weight on SSC11 at 48.9 Mb (see Additional files 3, 4, 5, 6 and 7: Figures S1, S2, S3, S4, and S5).

Among the SNPs within these regions, the most significant SNP across all traits was rs80935535 (*p* = 1.91E−10) on SSC7 at 34.8 Mb, which accounted for 13.2 % of the phenotypic variance for liver weight in F_2_ pigs. This SNP was also associated with heart weight (*p* = 1.48E−09) and explained 13.2 % of the phenotypic variance. The second most significant SNP was rs81318741 (*p* = 2.0E−10) on SSC2 at 0.9 Mb, which explained 8.6 % of the phenotypic variance for heart weight in F_2_ pigs (Table 2; Fig. 1).

### Meta-analysis of GWAS datasets

**Fig. 2:**
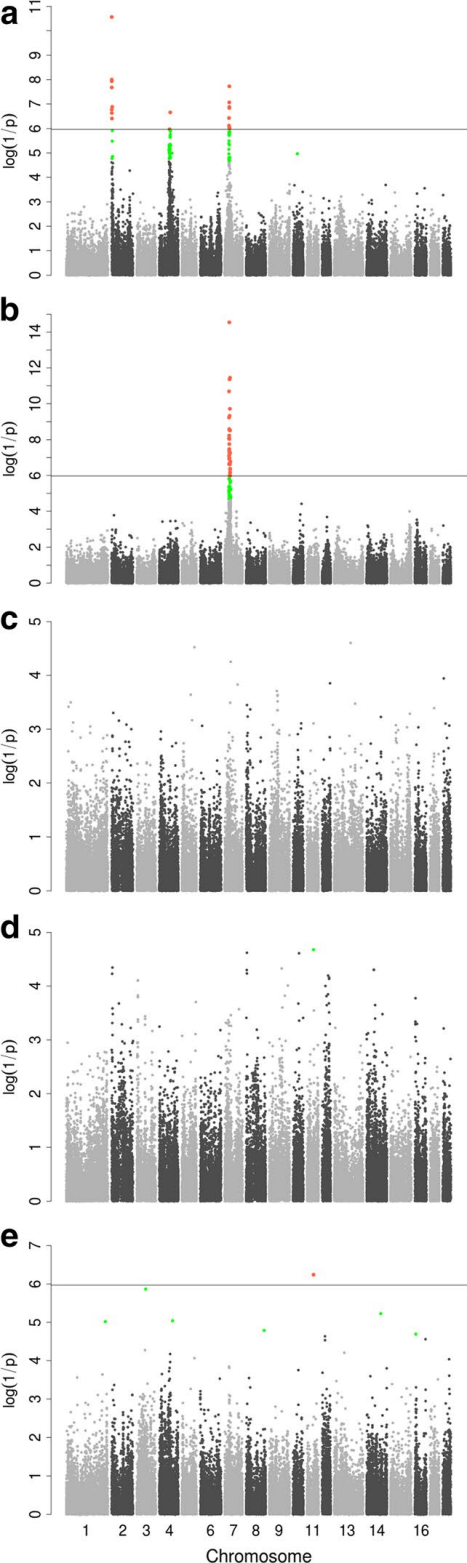
Meta-analysis of GWAS results for internal organ weights and carcass traits from five populations. Manhattan plots for GWAS of heart weight (**a**), liver weight (**b**), spleen weight (**c**), kidney weight (**d**) and carcass weight (**e**) from five populations. Meta-analysis of single-population GWAS was performed by combining the *p* values of the GWAS results from the five populations. In the Manhattan plots, negative log_10_ *p* values of the qualified SNPs are plotted against their genomic positions; the *red* and *green dots* represent SNPs that exceeded suggestive and 5 % genome-wide significance thresholds, respectively; *solid lines* indicate the 5 % genome-wide Bonferroni-corrected threshold

A meta-analysis of the single-population GWAS datasets was performed by combining Chi square statistics of the GWAS results from each of the five populations, separately for each SNP (Fig. 2 and Table S3 (Additional file 8: Table S3). The results confirmed most of the genome-wide significant QTL that were identified in the single-population GWAS. Among the confirmed SNPs, 23.2 % were detected with a higher significance level in the meta-analysis than in the single-population GWAS. Moreover, we detected four novel loci that reached suggestive significance on SSC1, 3, 8 and 16 for carcass weight, which were not detected in any of the single-population GWAS. This shows that a meta-analysis of GWAS results allows the discovery of novel QTL by combining detection signals across populations. Twenty-six suggestive QTL that were detected in the single-population analyses were missed in the meta-analysis. This finding indicates that the meta-analysis of GWAS datasets including even more different populations would contribute to discover more loci with moderate effects that would probably remain undetected because of population heterogeneity due to the limited number of individuals in the current populations. Therefore, single-population GWAS and meta-analysis of GWAS are complementary for the identification of QTL.

## Discussion

In this study, we detected 39 QTL for five traits in five experimental populations. Of these 39 QTL, nine reached the genome-wide significance level. Association signals were strongest for heart weight on SSC2, 4, 7 and for liver weight on SSC7 (Table 2; Fig. 1). Of these 39 QTL, eight confirmed previous reports from the pig QTL database (http://www.animalgenome.org/cgi-bin/QTLdb/SS/index). For example, the QTL for heart weight on SSC2 was identical to the QTL identified by Jeon et al. [26] and Wei et al. [27] and the QTL for liver heart on SSC7 was very similar to the QTL detected by Liu et al. [17] and Yue et al. [28].

These previous studies were mostly based on linkage analysis, which uses recent recombination events and thus, the mapping resolution is low (regions of more than 10 Mb and comprising hundreds of genes); in comparison, GWAS takes advantage of historical recombination events to reflect associations between markers and phenotypes, which results in a much higher resolution. Based on the rule of a LOD score cut-off of 2 [29], the confidence intervals of the regions that we identified on SSC2 in the F_2_ population were reduced to 0.3 Mb for heart weight (between 0.98 and 1.28 Mb) and 2.7 Mb for liver weight (between 34.7 and 37.4 Mb). Thus, GWAS is a powerful method to detect QTL that underlie complex traits or diseases and is currently applied for most major species.

### Comparison of our results with those of previous QTL mapping studies

Compared to our previous QTL mapping study based on microsatellite markers in the F_2_ intercross population, which identified 22 significant QTL [16], this GWAS based on high-density SNPs revealed only nine significant QTL. The likely reason is that the sample size is smaller in our study (928 individuals instead of 1028 individuals used in Ma et al. [16]), which results in reduced power and in more stringent significance thresholds, since in the Ma et al. GWAS a larger number of tested SNPs was used for Bonferroni correction. Moreover, QTL linkage mapping assumes that QTL alleles are alternatively fixed in each founder breed of the F_2_ intercross. The GWAS model adopted in our study only included additive effects, while the QTL mapping strategy applied in [16] included both additive and dominance effects, which may be another reason why fewer QTL were detected in the GWAS analysis. To test the hypothesis that the current study may have missed QTL because of dominance, we performed a dominance GWAS using PLINK (v1.07) for kidney weight in the F_2_ population. We compared the significance of average phenotype differences between heterozygous and homozygous individuals at the tested SNP. The Manhattan plot of the dominance GWAS for kidney weight showed that among the three detected QTL, there were one genome-wide QTL and two suggestive QTL (see Additional file 9: Figure S6). Compared to the additive model that detected no QTL for kidney weight, the dominance model led to the identification of three novel QTL for this trait. Thus, some of the QTL that were missed in our GWAS were dominant.

The GWAS reported here confirmed two QTL that were identified in our previous linkage analysis [16] on the F_2_ population: one on SSC2 for heart weight and one on SSC4 for carcass weight; in addition, we detected four novel QTL, one for heart weight, one for liver weight and two for spleen weight. It should be noted that the QTL for liver weight on SSC7 was also identified by Liu et al. [17] within a 9.7 Mb region (between 31.24 and 41.00 Mb) but the GWAS allowed us to reduce the confidence interval to 2.7 Mb. This implies that linkage analysis and GWAS complement each other to detect genetic variants for traits of interest.

### Genetic homologies and differences between populations

Using the F_2_ pig intercross, two major QTL were detected for heart weight on SSC2 at 0.9 Mb and on SSC7 at 34.7 Mb. These two QTL were also identified with the linkage mapping strategy [16]. In contrast, no signal was detected for these traits when using the DLY commercial or Erhualian populations (that have founders similar to those of the F_2_ population), which implies that these two QTL were fixed in the founders of these two breeds or were segregating at a sufficiently low frequency such that they were not detected, or were false positives. Thus, this QTL could only be detected by using an intercross between these two breeds. Allele frequencies of the top SNP on SSC7 for heart weight were equal to 0.78 and 0.00 in the DLY and Erhualian breeds, respectively, which means that it was fixed in the Erhualian population. Of course, LD patterns between causative mutation and detected SNP can differ between populations and a much more complex LD structure may exist in the DLY hybrid population. Furthermore, in the Sutai population, a suggestive QTL for heart weight on SSC2 was replicated when analyzing the F2 population, which indicates that the QTL effect is homogeneous between the F2 and Sutai populations and thus that a common mutation is present. In the Laiwu population, a novel QTL was detected on SSC4 at 77.2 Mb but no association signal was identified for this region in any other population. Since this QTL was only observed in the Chinese specialized local breed, it indicates that the genetic architecture that underlies heart weight differs between the five populations studied here. Chinese native breeds cover about one third of the world’s genetic pig resources and since their genetic diversity is higher than that of commercial breeds, they increase the genetic variability of interbreed crosses.

Possible reasons for the differences observed between GWAS across populations [30] could be that: (a) the collective effect of many rare mutations plays a substantial role in complex phenotypes; (b) a common disease in unrelated affected individuals is caused by many (hundreds or even thousands) different rare severe mutations in the same gene; (c) the penetrance of a mutation differs between individuals; (d) mutations in different genes of the same or related pathway result in the same disorder; and (e) gene by gene and gene by environment interactions lead a causative mutation to have different genetic effects between populations [30]. Therefore, understanding the genetic heterogeneity of traits will benefit personal medicine and therapy.

### Pleiotropic QTL

On SSC7, a pleiotropic QTL (top SNP rs80935535 at 34.8 Mb) was detected for both heart weight and liver weight in F_2_ pigs. Allele A inherited from the Erhualian breed increases the weights of liver and heart. Pleiotropy can be explained by different mechanisms: (1) alternative splicing events and alternate start or stop codons that can lead to different proteins and, thus, impact different phenotypes; (2) the gene network theory, which assumes that a key driver gene in a gene interaction network can impact several pathways and, thus, if this gene is mutated, pleiotropy is observed; and (3) a gene that has the same effect across multiple tissues [31]. A highly significant correlation (r^2^ = [0.59 − 0.67]) between heart weight and liver weight was observed for the F_2_ pigs (see Additional file 1: Table S1), which suggests that the development of these two organs may be under the control of the same gene pathway. Previous studies showed that traits that are under the influence of pleiotropic QTL tend to cluster together or to be highly correlated, such as overall body size [32] and adiposity [33]. The weight of organs can be expressed as a percentage of the body weight that varies with overall body size. Thus, the genetic mechanisms that are responsible for the weight of organs could be interrelated in some ways.

### Plausible candidate genes in the identified QTL regions

To identify potential candidate genes that may impact the weight of organs, we examined the functions of the genes that are located within the confidence intervals of genome-wide significant QTL. Three interesting candidate genes were found on the basis of their position, functional annotation and reported expression patterns. The region, which contains a QTL for heart weight in the F_2_ and Sutai populations and is located at the proximal end of SSC2, harbors the *IGF2* (*insulin*-*like growth factor II*) gene, which is involved in a paternally expressed QTL with major imprinting effects on muscle mass, fat deposition, backfat thickness and heart size [26]. A single nucleotide substitution in intron 3 of the *IGF2* gene was shown to have major effects on skeletal muscle in pigs since it eliminates a binding site for a repressor and results in a threefold up-regulation of *IGF2* [26]. Since *IGF2* is a paternally expressed gene, we hypothesized that the QTL detected for heart weight may also be imprinted. To verify this, we conducted imprinting-linkage mapping (i-QTL) versus a non-imprinting test [34] at the top SNP (rs81318741) in the F_2_ population. The test model was formulated as:

1 $${\text{y}} = c_{a} \alpha + e$$

and

2 $${\text{y}} = c_{a} \alpha + c_{I} \beta + e$$

where $c_{a}$ is the probability that F_2_ individuals are of Duroc origin, and $c_{I}$ is the difference between the probabilities of the heterozygous F_2_ individuals to have Duroc–Erhualian and Erhualian–Duroc phases. Imprinting tests were performed by comparing the probability of the model with and without imprinting effect. QTL detected under the model with imprinting effect were more significant than those detected under the model without imprinting effect (*p* value = 5.68E−06). Here, we confirmed that the QTL detected for heart weight was imprinted. However, identification of the causative mutation for heart weight needs further investigation.

SSC7 encompasses one major QTL for heart weight in the F_2_ population and for liver weight in the F_2_ and Laiwu populations. Within an interval of 5 Mb around the confidence interval ranging from 34.7 to 37.4 Mb, *GRM4* (*glutamate receptor, metabotropic 4*) was identified as a strong candidate gene for this QTL based on its biological function and position, which was exactly at the same position as the most significant SNP (rs80935535, 34.8 Mb). *GRM4* is expressed during the differentiation of embryonic stem cells into myocardial cells and its expression declines with maturation of the myocardial cells [35]. *HMGA1* (*high mobility group AT*-*hook 1*) is another candidate gene adjacent to *GRM4* on SSC7 for heart weight and liver weight. *HMGA1* has been reported to be associated with growth in pigs Liu et al. [16], and [36, 37] also considered this gene as the prime biological candidate for carcass traits and internal organ weights.

## Conclusions

In conclusion, a total of 39 loci on 15 chromosomes were identified. The results confirmed some of the previously identified QTL but also identified several novel QTL for these traits. Besides, we identified three potential candidate genes, *IGF2* for heart weight located on SSC2, *GRM4* for heart weight and liver weight on SSC7 and *HMGA1* for heart and liver weight on SSC7. We also compared genetic homologies and differences between these traits in different populations.

## Additional files

10.1186/s12711-015-0168-7 Results of the correlation analysis for traits studied in five populations. This table contains the estimated correlations and 95 % confidence intervals between traits for F_2_ pigs (Table S1A), Sutai pigs (Table S1B), Laiwu pigs (Table S1C), Erhualian pigs (Table S1D) and DLY pigs (Table S1E).
10.1186/s12711-015-0168-7 Suggestive significant SNPs for the weights of four internal organs and carcass in five populations. A total of 248 suggestive significant SNPs were identified in our study, including 91 for heart weight, 125 for liver weight, 6 for spleen weight, 12 for kidney weight and 14 for carcass weight.
10.1186/s12711-015-0168-7 Single-population GWAS results for heart weight. Manhattan plots of GWAS results for heart weight in Sutai pigs (A), Erhualian pigs (B) and commercial pigs (C). In the Manhattan plots, negative log_10_ *P* values of the qualified SNPs were plotted against their genomic positions. Green dots are SNPs that surpass the suggestive significance level; red dots are the SNPs that surpass the genome-wide significance level; solid lines indicate the 5 % genome-wide Bonferroni-corrected threshold.
10.1186/s12711-015-0168-7 Single-population GWAS results for liver weight. Manhattan plots of GWAS results for liver weight in Sutai pigs (A), Erhualian pigs (B) and commercial pigs (C). In the Manhattan plots, negative log_10_ *P* values of the qualified SNPs were plotted against their genomic positions. Green dots are SNPs that surpass the suggestive significance level; red dots are the SNPs that surpass the genome-wide significance level; solid lines indicate the 5 % genome-wide Bonferroni-corrected threshold.
10.1186/s12711-015-0168-7 Single-population GWAS results for spleen weight. Manhattan plots of GWAS results for spleen weight in F_2_ pigs (A), Sutai pigs (B), Erhualian pigs (C) and Laiwu pigs (D) and commercial pigs (E). In the Manhattan plots, negative log_10_ *P* values of the qualified SNPs were plotted against their genomic positions. Green dots are SNPs that surpass the suggestive significance level; red dots are the SNPs that surpass the genome-wide significance level; solid lines indicate the 5 % genome-wide Bonferroni-corrected threshold.
10.1186/s12711-015-0168-7 Single-population GWAS results for kidney weight. Manhattan plots of GWAS results for kidney weight in F_2_ pigs (A), Sutai pigs (B), Erhualian pigs (C) and Laiwu pigs (D). In the Manhattan plots, negative log_10_ *P* values of the qualified SNPs were plotted against their genomic positions. Green dots are SNPs that surpass the suggestive significance level; red dots are the SNPs that surpass the genome-wide significance level; solid lines indicate the 5 % genome-wide Bonferroni-corrected threshold.
10.1186/s12711-015-0168-7 Single-population GWAS results for carcass weight. Manhattan plots of GWAS results for carcass weight in F_2_ pigs (A), Sutai pigs (B), Erhualian pigs (C) and Laiwu pigs (D) and commercial pigs (E). In the Manhattan plots, negative log_10_ *P* values of the qualified SNPs were plotted against their genomic positions. Green dots are the SNPs that surpass the suggestive significance level; red dots are the SNPs that surpass the genome-wide significance level; solid lines indicate the 5 % genome-wide Bonferroni-corrected threshold.
10.1186/s12711-015-0168-7 Suggestive significant SNPs for the traits studied in the meta-analysis of GWAS datasets. A total of 148 suggestive significant SNPs were identified. Most of these SNPs were within the QTL regions that were identified by single-population GWAS. Four novel QTL associated with carcass weight were identified in the meta-analysis, which are highlighted in yellow. ** = genome-wide significance level and * = suggestive significance level.
10.1186/s12711-015-0168-7 Results of the dominance GWAS for kidney weight in the F_2_ population. In the Manhattan plots, negative log_10_ *P* values of the qualified SNPs were plotted against their genomic positions; the red and green dots represent the SNPs that exceeded the genome-wide significance and suggestive significance thresholds, respectively; solid lines indicate the 5 % genome-wide Bonferroni-corrected threshold.
