# Supplementary figures and images for: Multi-breed genome-wide association study reveals novel loci associated with the weight of internal organs
Source: Genet Sel Evol. 2015 Nov 17;47:87. doi: 10.1186/s12711-015-0168-7 (PMC4647478; doi:10.1186/s12711-015-0168-7)

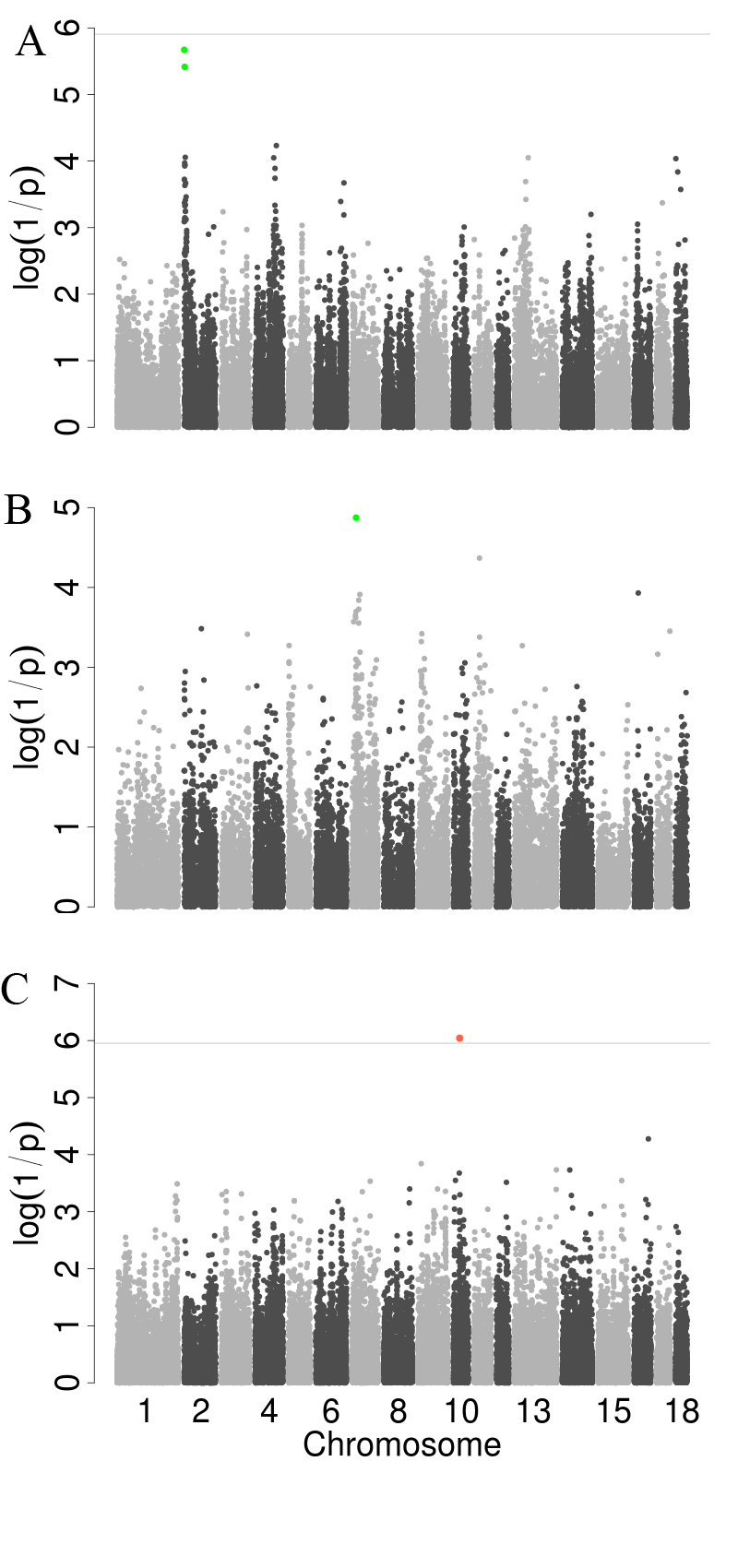

Supplement: Supplementary file 3 — 10.1186/s12711-015-0168-7 Single-population GWAS results for heart weight. Manhattan plots of GWAS results for heart weight in Sutai pigs (A), Erhualian pigs (B) and commercial pigs (C). In the Manhattan plots, negative log10 P values of the qualified SNPs were plotted against their genomic positions. Green dots are SNPs that surpass the suggestive significance level; red dots are the SNPs that surpass the genome-wide significance level; solid lines indicate the 5 % genome-wide Bonferroni-corrected threshold. [file 12711_2015_168_MOESM3_ESM.png]

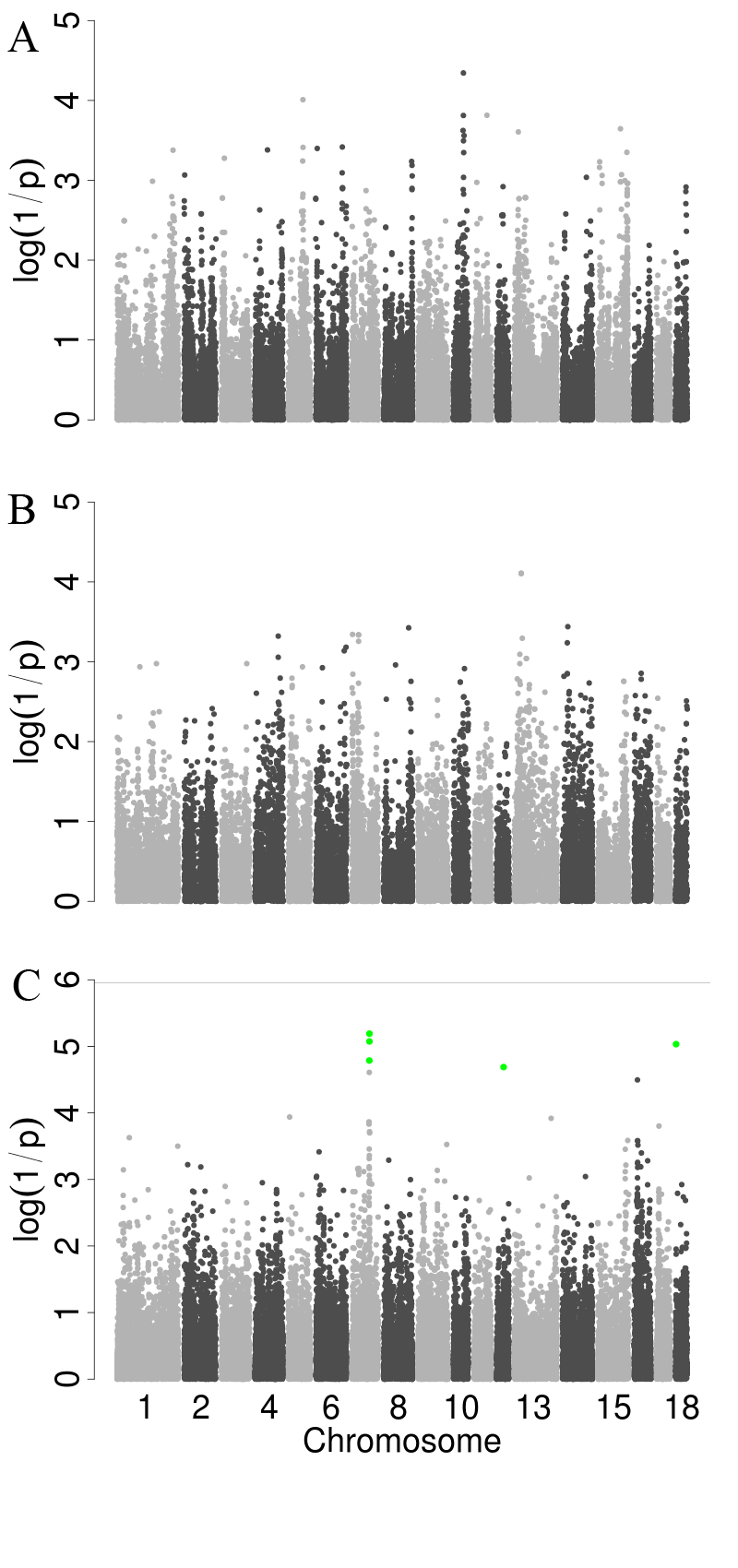

Supplement: Supplementary file 4 — 10.1186/s12711-015-0168-7 Single-population GWAS results for liver weight. Manhattan plots of GWAS results for liver weight in Sutai pigs (A), Erhualian pigs (B) and commercial pigs (C). In the Manhattan plots, negative log10 P values of the qualified SNPs were plotted against their genomic positions. Green dots are SNPs that surpass the suggestive significance level; red dots are the SNPs that surpass the genome-wide significance level; solid lines indicate the 5 % genome-wide Bonferroni-corrected threshold. [file 12711_2015_168_MOESM4_ESM.png]

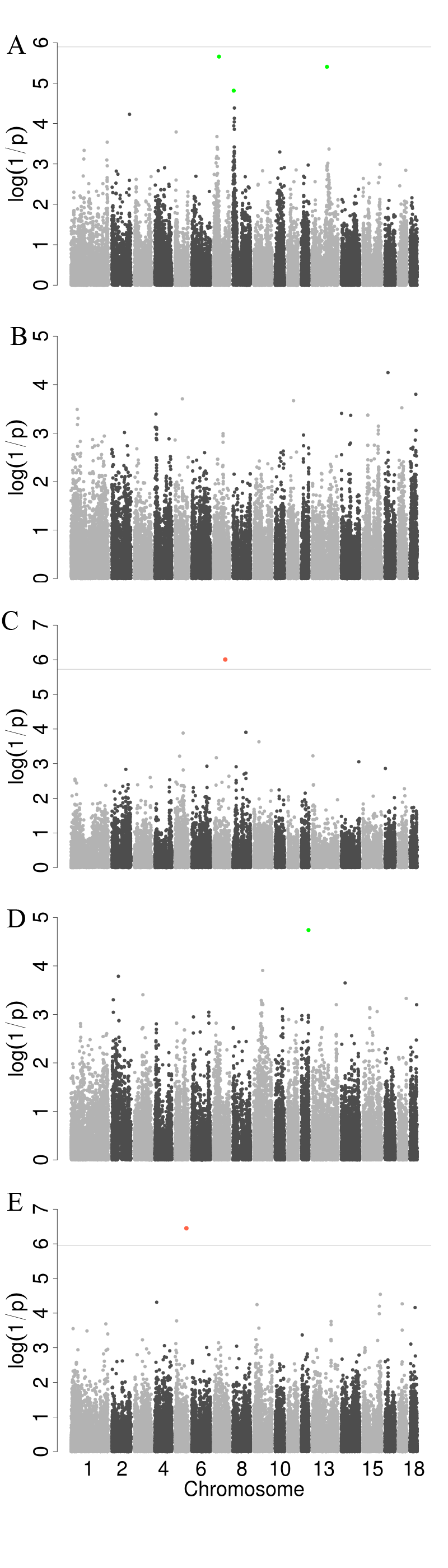

Supplement: Supplementary file 5 — 10.1186/s12711-015-0168-7 Single-population GWAS results for spleen weight. Manhattan plots of GWAS results for spleen weight in F2 pigs (A), Sutai pigs (B), Erhualian pigs (C) and Laiwu pigs (D) and commercial pigs (E). In the Manhattan plots, negative log10 P values of the qualified SNPs were plotted against their genomic positions. Green dots are SNPs that surpass the suggestive significance level; red dots are the SNPs that surpass the genome-wide significance level; solid lines indicate the 5 % genome-wide Bonferroni-corrected threshold. [file 12711_2015_168_MOESM5_ESM.png]

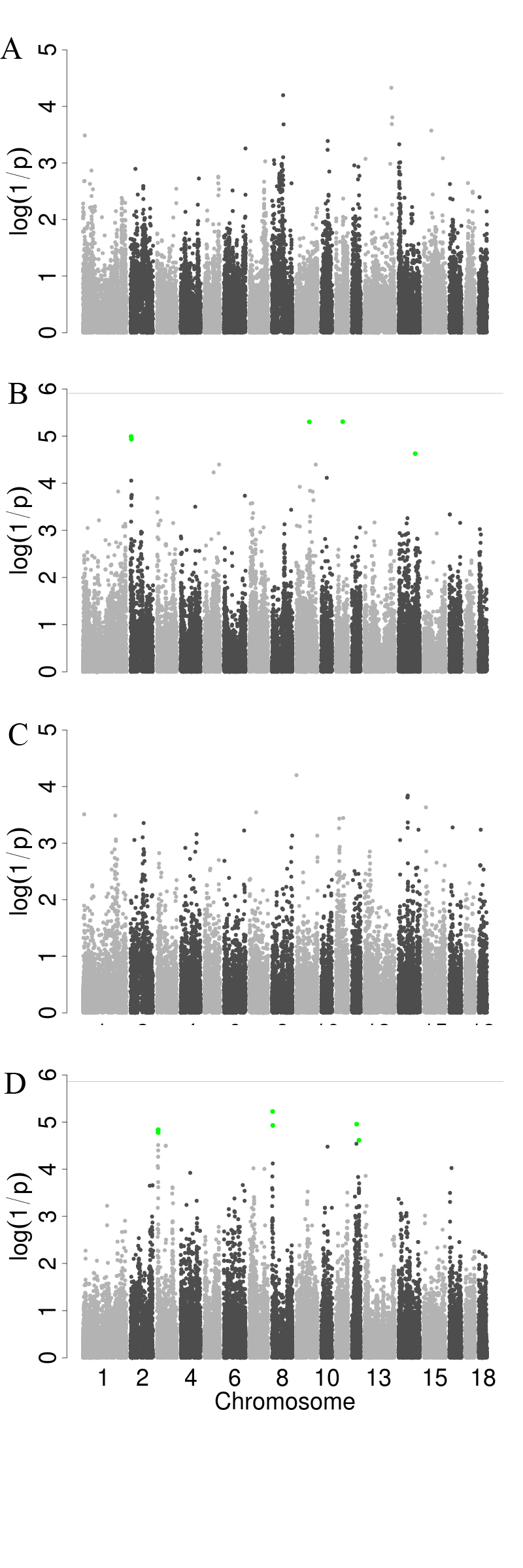

Supplement: Supplementary file 6 — 10.1186/s12711-015-0168-7 Single-population GWAS results for kidney weight. Manhattan plots of GWAS results for kidney weight in F2 pigs (A), Sutai pigs (B), Erhualian pigs (C) and Laiwu pigs (D). In the Manhattan plots, negative log10 P values of the qualified SNPs were plotted against their genomic positions. Green dots are SNPs that surpass the suggestive significance level; red dots are the SNPs that surpass the genome-wide significance level; solid lines indicate the 5 % genome-wide Bonferroni-corrected threshold. [file 12711_2015_168_MOESM6_ESM.png]

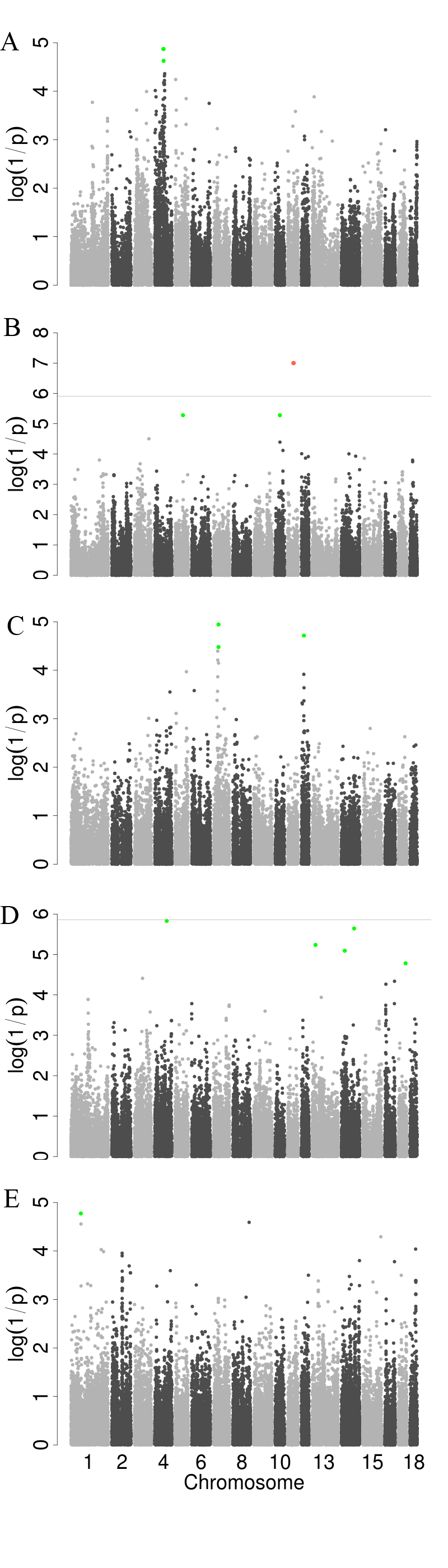

Supplement: Supplementary file 7 — 10.1186/s12711-015-0168-7 Single-population GWAS results for carcass weight. Manhattan plots of GWAS results for carcass weight in F2 pigs (A), Sutai pigs (B), Erhualian pigs (C) and Laiwu pigs (D) and commercial pigs (E). In the Manhattan plots, negative log10 P values of the qualified SNPs were plotted against their genomic positions. Green dots are the SNPs that surpass the suggestive significance level; red dots are the SNPs that surpass the genome-wide significance level; solid lines indicate the 5 % genome-wide Bonferroni-corrected threshold. [file 12711_2015_168_MOESM7_ESM.png]

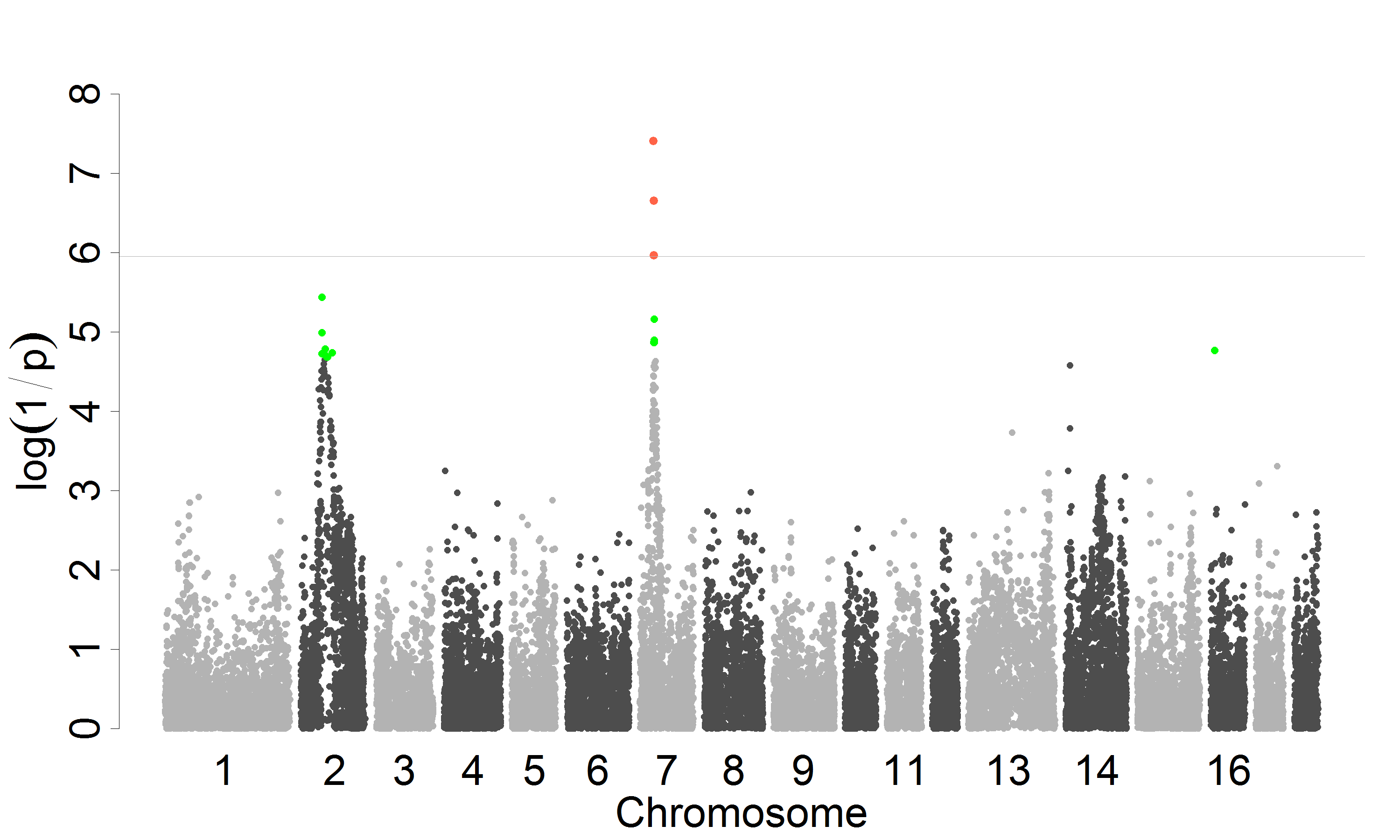

Supplement: Supplementary file 9 — 10.1186/s12711-015-0168-7 Results of the dominance GWAS for kidney weight in the F2 population. In the Manhattan plots, negative log10 P values of the qualified SNPs were plotted against their genomic positions; the red and green dots represent the SNPs that exceeded the genome-wide significance and suggestive significance thresholds, respectively; solid lines indicate the 5 % genome-wide Bonferroni-corrected threshold. [file 12711_2015_168_MOESM9_ESM.png]
